# Sex and Urban–Rural Differences in the Relationship between Childhood Sexual Abuse and Mental Health among Chinese College Students

**DOI:** 10.3390/ijerph19159225

**Published:** 2022-07-28

**Authors:** Rudong Zhang, Yun Liang, Wenzhen Cao, Leixiao Zeng, Kun Tang

**Affiliations:** 1 Vanke School of Public Health, Tsinghua University, Beijing 100083, China; zhangrudong@mail.tsinghua.edu.cn (R.Z.); liangy21@mails.tsinghua.edu.cn (Y.L.); 2 School of Public Health and Management, Wenzhou Medical University, Wenzhou 325035, China; caowz@pku.edu.cn; 3 School of Journalism and Communication, Renmin University of China, Beijing 100872, China; s19500262@ucass.edu.cn

**Keywords:** childhood sexual abuse, mental health, college students, suicide attempts, sex

## Abstract

This study aimed to reveal sex and urban–rural differences in the association between childhood sexual abuse (CSA) and mental health among Chinese college students. The study used data from the “National College Student Survey on Sexual and Reproductive Health 2019”, a cross-sectional study conducted in all 31 provinces of mainland China. Weighted logistic regression analysis was conducted to determine the association between CSA (noncontact CSA, contact CSA, and penetrative CSA) and mental health (suicide attempts and mental disorders). Among 49,728 students, 39.42% of the male participants and 43.55% of the female participants had ever experienced CSA. According to the OR results of logistic regression analysis, compared to females, males in the contact CSA group (AOR: 3.49, 95% CI: 1.95–6.23) and the penetrative CSA group (AOR: 8.79, 95% CI: 3.15–24.52) had higher odds of suicide attempts. Participants from rural and suburban areas that were categorized in the penetrative CSA group were more likely to report suicide attempts (rural: AOR: 4.01, 95% CI: 1.51–10.62, suburban AOR: 4.86, 95% CI: 2.52–9.36) and mental disorders (rural: AOR: 4.01, 95% CI: 1.51–10.62, suburban: AOR: 4.86, 95% CI: 2.52–9.36). In conclusion, the findings revealed a high prevalence of CSA in both sexes and reported that males are more vulnerable to the adverse effects of CSA. In addition, we also found that undergraduates growing up in rural and suburban areas are more vulnerable to the adverse psychological effects of CSA. Policymakers should pay more attention to this vulnerable population and implement effective measures to alleviate mental trauma.

## 1. Introduction

Childhood sexual abuse (CSA) remains a serious public health issue worldwide. There are different definitions of CSA. For example, the United States Centers for Disease Control and Prevention defines CSA as “the involvement of a child (person less than 18 years old) in sexual activity that violates the laws or social taboos of society” [1]. It includes behaviours such as fondling, penetration, and exposing a child to other sexual activities (CDC, n.d.) [2]. According to the United Nations Children’s Fund, sexual abuse is the actual or threatened physical intrusion of a sexual nature, whether by force or under unequal or coercive conditions, perpetrated by aid workers against the children and families they serve. [3]. To provide a more comprehensive investigation of the impact of CSA, this study referenced a similar definition of CSA based on our earlier article but included verbal sexual harassment [4].

CSA is a widespread occurrence among boys and girls hailing from both rural and urban areas and across a range of cultures and socioeconomic backgrounds [5]. In recent decades, several studies conducted in different countries have investigated the prevalence of CSA and identified a generally high rate; 1 in 5 women and 1 in 13 men reported experiencing CSA worldwide [6]. In China, the prevalence of CSA in males is higher than the global rate. A literature review of epidemiological studies on CSA in China indicated that the prevalence of CSA among females was 14.2~25.5%, and the prevalence of CSA among males was 10.2~23.0%.

A history of CSA is associated with not only short-term consequences, such as symptoms characterized in child clinical samples, but also long-term emotional consequences, including anxiety, fear, and suicidal ideation [7,8]. Survivors of CSA are more likely to report an increased risk of mental disorders, and a study with a nationally representative sample of 9282 adults in the United States showed that CSA was associated with 26% to 32% of adult psychiatric disorders [9]. A birth cohort study in New Zealand provided similar results [10]. In addition, reports indicate that CSA is linked to high levels of suicide attempts. The survivors of CSA have a two- to three-fold risk of suicidal ideation and a three- to four-fold risk of attempted suicide as opposed to those who did not experience CSA [10]. Additionally, in China, limited studies have suggested that CSA is associated with mental health outcomes. A study showed that in rural China, adults with a history of CSA had significantly higher rates of depression and suicidal attempts [11]. Similarly, a national survey reported that adults who suffered from CSA had a high risk of psychological distress [12].

Although there are many scientific findings on CSA and mental health in China, on the one hand, most studies were prone to focus on female populations [13,14,15] and disclose the prevalence and impact of CSA on females [16]. As such, it can be deduced that unequal attention has been given to male CSA occurrences in China. Underreporting of CSA among males could be justified by societal prejudices and phenomena such as toxic masculinity that may prevent survivors from actively seeking help and treatment, leading to prolonged suffering and considerable stress. On the other hand, China’s economy has experienced a rapid boom, and urbanization has promoted the massive migration of rural residents to cities in recent years. Migrant parents have to leave their children in their impoverished villages due to the strict household registration system and high expenditure of raising children in cities, resulting in the absence of parental discipline. This means that children in rural areas cannot attain enough social and economic protection in a timely manner similar to urban children [17]. Furthermore, urban–rural differences, which are often severe and intractable, are often perpetrators of social and health inequalities [18]. Comprehensive sexuality education and psychological interventions are particularly scarce in rural and remote areas. It is therefore of interest to explore the discrepancies between CSA and mental health both in females and males and among rural, suburban, and urban areas. Our study depicted the prevalence of CSA in a large sample size of college students from across all 31 provinces of China and analysed the sex and urban–rural differences pertaining to the associations with CSA and mental health. Drawing from the theory of social determinants of health [19], which puts much emphasis on demographic and socioeconomic factors on health, this study aims to explore the following questions: (1) Is there a difference in the relationships between childhood sexual abuse and mental health between college students who come from rural versus suburban areas versus urban areas? (2) Is there a difference in the relationships between childhood sexual abuse and mental health between male college students and female students?

## 2. Materials and Methods

### 2.1. Participants

The current study was a secondary data analysis using data from a cross-sectional study conducted across all 31 provinces of mainland China from November 2019 to February 2020. The data were collected through the China Youth Network (CYN), the largest youth volunteer organization for sexual and reproductive health education in China, with the objective of providing a more representative analysis of the sexual behaviours and sexual attitudes among Chinese college students. The study was approved by the Tsinghua University Institutional Review Board (IRB) (Project No: 20190083).

Through multistage sampling along with references from the List of Institutions of Higher Learning released by China’s Ministry of Education in 2018, 241 higher learning institutions were selected after adjustment in accordance with population density and different levels of educational institutions in China. The survey questionnaires were distributed to students through designated persons of contact in each institution.

Due to the snowball effect that accelerated the distribution of the online survey, responses from 55,757 university and vocational college students were collected. Of those, 1177 (2%) respondents either did not properly complete the attention check questions (Supplemental Material File S1) or did not provide informed consent for the release of their information. After excluding these respondents, a total of 54,580 students were regarded as having valid questionnaires. Then, the surveys were limited by age to isolate an ideal range of undergraduate students aged 16–24 years old, the major age range of Chinese college students. After excluding data from master’s and doctoral students (excluding 3885 participants) and restricting the age range (excluding 867 participants), the unweighted crude sample size analysed in our study was 49,728 participants (16,999 males, 32,799 females).

The original questionnaire consisted of 114 questions across four domains: sexual and reproductive health knowledge and attitudes, gender/same-sex interactions, personal health and behavioural conditions, and basic personal and family conditions. The questions utilized in our study are presented in Supplemental material (Chinese and English versions).

### 2.2. Childhood Sexual Abuse

The types of CSA experiences outlined included (1) online verbal sexual harassment; (2) verbal sexual harassment in real-life situations; (3) being forced to expose their breasts or genitals; (4) having one’s private parts (breasts or genitals) touched or fondled involuntarily; (5) being subjected to involuntary oral intercourse; and (6) being subjected to involuntary vaginal or anal intercourse. The types of CSA that participants had suffered from were consequently categorized into three groups: noncontact CSA (i.e., verbal sexual harassment), contact CSA (i.e., unwanted exposure of genitals, touching or fondling genitals), and penetrative CSA (i.e., penetrative oral, vaginal, or anal intercourse) in accordance with the severity of CSA.

Participants were asked whether they had ever experienced any of the abovementioned types of sexual abuse during their childhood and puberty stages. Respondents who gave an affirmative response to one or more types of CSA were defined as having been exposed to CSA. In addition, it was deduced that the proportion of participants who were categorized into the same groups (noncontact, contact, and penetrative CSA) based on the different categories of CSA was close.

### 2.3. Mental Health

The prevalence of suicide attempts was investigated through a multiple-choice question (“Have you ever attempted suicide or tried to take your own life?”; optional answers were as follows: no suicidal ideation or attempts; only suicidal ideation; one suicide attempt; more than one suicide attempt). The participants were categorized into “Yes” or “No” in accordance with their response to whether they had ever attempted suicide.

Participants’ mental disorders, as the outcome variable, were measured by the following question: “Have you ever been clinically diagnosed with any type of mental disorder?” The options for mental disorders include depression, anxiety, bipolar disorder, obsessive-compulsive disorder, anorexia or bulimia, attention deficit hyperactivity disorder, phobia, posttraumatic stress disorder, personality disorders, schizophrenia, and substance abuse. As each separate mental disorder has a very low prevalence, we integrated them as a new variable to better explore the associations. Respondents who self-reported any of the above mental disorders were classified as having any mental disorders.

### 2.4. Covariates

The covariates included age (“16–18 years”, “19–21 years”, or “22–24 years”), sex (“male” or “female”), socioeconomic status, and behavioural risk factors.

#### 2.4.1. Socioeconomic Status

Socioeconomic status was measured by the following: (1) Parental education level: (“unknown”, “primary school and below”, “middle school or high school”, or “vocational school or above”). In the study, we defined the “parental education level” as the higher educational attainment of one parent or guardian of respondents. (2) Monthly expenditure: Average monthly expenditures of the participants in the past 12 months (“Less than CNY 1500” and “CNY 1500 or more”). We approximately draw the median monthly expenditure of the respondents as the cut-off value. Based on international exchange rates, CNY 1500 yuan is equivalent to USD 240. (3) Family structure: (“complete family” or “the others”). Respondents belonging to a family with no parental divorce, bereavement, or separation during their childhood and adolescence were defined as having a “complete family”. (4) Residential environment: type of residential environment (“urban”, “suburban”, or “rural”). The residential environment was assessed through the urbanization level of residential areas of respondents prior to attending college.

#### 2.4.2. Behavioural Risk Factors

Behavioural risk factors included cigarette and alcohol use. Previous literature has established that cigarette and alcohol use are associated with suicidal behaviour among adolescents [20,21]. Cigarette use was sorted into groups such as “never”, “0–5 cigarettes/day”, and “> 5 cigarettes/day” based on daily cigarette intake. For alcohol use, participants were categorized into “non-drinker (never drink or seldom)”, “occasional drinker (less than once per week)”, and “frequent drinker (at least once per week)”.

### 2.5. Statistical Analysis

All statistical analyses were examined using Stata version 14.0 (StataCorp, College Station, TX, USA). Two-sided *p-*values were considered significant at *p* < 0.05. Weights were applied to account for undersampling and oversampling and were adjusted according to school locations (east, central, west), school types (vocational college, college), grades, and sex in different provinces with reference to the Educational Statistics Yearbook of China 2018 [22]. The weighted data are nationally representative and appropriate for making substantiated decisions on health care policies concerning the sexual and reproductive health of college students.

First, descriptive analyses were conducted to describe the prevalence of reported CSA between both sexes. Additionally, we performed descriptive analyses to profile the distribution of mental health and other sociodemographic variables in the study population stratified by the severity of CSA. All categorical data were compared using chi-square tests and expressed as proportions. Second, multiple logistic regression models were used to examine associations between CSA and mental health variables stratified by sex and urban–rural factors. To account for clustering by the university level, standard errors of the estimates were estimated using the clustered robust variance method. Diagnostic analyses indicated that all other conditions for regression models, including normality and linearity assumptions, were met. Adjusted odds ratios, 95% confidence intervals, and *p*-values were calculated and reported.

## 3. Results

Table 1 shows the prevalence of different types of CSA experiences among participants. Of the 49,728 participants included in the analyses, 41.98% had experienced CSA, including 39.90% of male participants and 43.84% of female participants. Upon further categorization, 28.64% of participants had experienced noncontact CSA, 11.07% had experienced contact CSA, and 2.27% had experienced penetrative CSA. According to the chi-square test, the severity of CSA experience differed between males and females (*p* < 0.001). Female participants experienced more contact CSA, which included forced exposure of private parts (6.27% of males vs. 12.71% of females, *p* < 0.001) and involuntary touching or fondling of private parts (6.71% of males vs. 13.14% of females, *p* < 0.001). For other CSA experiences, there were no significant differences between males and females.

The basic social, environmental, and lifestyle characteristics of our participants are shown in Table 2. Compared to the non-CSA group, the severity of CSA experienced by the participants was more strongly associated with older age, having an incomplete family, having high monthly expenditures, and having a history of smoking and drinking. A total of 2.37% of participants in the non-CSA group reported suicide attempts, while the prevalence of suicide attempts among participants from the noncontact CSA group, contact CSA group, and penetrative CSA group was 4.47%, 7.98%, and 15.02%, respectively. The prevalence of participants with mental diseases in the non-CSA group, noncontact CSA group, contact CSA group, and penetrative CSA group was 5.24%, 9.78%, 13.48%, and 18.02%, respectively. Table 2 also describes the prevalence of each mental disorder. We found that as the severity of CSA increased, the prevalence of both suicide attempts and mental diseases increased correspondingly (*p* < 0.001).

Table 3 and Table 4 present the logistic regression results stratified by sex and residential environment. The prevalence of suicide attempts among females and males was 4.96% and 2.58%, respectively. A total of 8.63% of females and 6.75% of males had ever suffered from mental disorders. For residential environments, the prevalence of suicide attempts among rural, suburban, and urban areas was 2.64%, 3.57%, and 4.58%, respectively. The prevalence of mental disorders among rural, suburban, and urban areas was 4.78%, 6.55%, and 9.95%, respectively.

Table 3 presents the associations between suicide attempts and mental disorders with different CSA experiences, stratified by sex. As the severity of CSA increased, the odds of suicide attempts and mental disorder prevalence increased in both the male and female groups. Moreover, males belonging to the contact CSA group (AOR: 3.49, 95% CI: 1.95–6.23) and penetrative CSA group (AOR: 8.79, 95% CI: 3.15–14.52) had higher odds of suicide attempt than females in the contact CSA group (AOR: 2.55, 95% CI: 1.88–3.47) and penetrative CSA group (AOR: 3.97, 95% CI: 2.51–6.26). However, this trend was not replicated with respect to mental disorders. The crude model revealed similar associations between suicide attempts and mental disorders across different CSA experiences.

Table 3 presents the associations between suicide attempts and mental disorders with different CSA experiences, stratified by the residential environment. The odds of suicide attempt prevalence increased in all three district groups (rural, suburban, and urban) when the severity of CSA experienced by participants increased. Similarly, in the penetrative CSA group, participants from rural (AOR: 8.69, 95% CI: 3.10–24.36) and suburban (AOR: 9.73, 95% CI: 3.29–28.78) areas had higher odds of suicide attempt. In contrast, the odds ratio of suicide attempts from the penetrative CSA category was found to be lower in urban areas (AOR: 2.70, 95% CI: 1.71–4.27). With regard to mental diseases, participants from rural (AOR: 4.01, 95% CI: 1.51–10.62) and suburban areas (AOR: 4.86, 95% CI: 2.52–9.36) with penetrative CSA experience had higher odds of suicide attempt. However, in urban areas, the odds ratio of the penetrative CSA group with mental disorders was lower (AOR: 2.36, 95% CI: 1.59–3.50). The relationships obtained by comparing the crude odds ratios were consistent with the adjusted odds ratios.

## 4. Discussion

The study included a large sample from all 31 provinces in mainland China and is one of the largest studies investigating the associations between the severity of CSA and mental health among Chinese college students as well as sex and urban–rural differences in these relationships. Our findings suggested that CSA was a major public health concern and that nearly 40% of Chinese college students experienced CSA. Those who experienced CSA were more likely to report suicide attempts and mental disorders. We also found a possible dose–response relationship, with a trend of greater severity of CSA corresponding to higher levels of harm in terms of mental disorders and suicide attempts. Compared with females, males with a history of contact and penetrative CSA were more likely to report suicide attempts. Participants from rural or suburban areas suffering from contact and penetrative CSA had a significantly higher risk of mental disorders and suicide attempts.

The prevalence of CSA in our study was reported as 41.98% (39.42% in males and 43.55% in females), which is significantly higher than other studies ranging from 10.1% to 24% across China and abroad [23,24,25]. Indeed, the possible reason for such a great discrepancy may be attributed to the different ethnic backgrounds, data gathering methodologies, demographic characteristics, and the main contributing factor of varying CSA definitions. In our study, we used a broader definition of CSA that included verbal sexual harassment online and in real-life situations (also defined as noncontact sexual abuse) to better investigate the dose–response relationship. When our study excluded the noncontact sexual abuse category, the rate of CSA decreased to 10.77% for males and 14.36% for females, which was similar to the results of a previous meta-analysis on 27 Chinese prevalence studies (10.5% for contact and penetrative sexual abuse) and a recent study with a large population sample (10.3% for contact and penetrative sexual abuse) [6,26]. Interestingly, our results indicated that the prevalence of contact and penetrative CSA was relatively lower than comparable international estimates. We attempted to explain this phenomenon in terms of cultural context and policy. Due to the conventional Confucian moral values of restraint, most Chinese people embrace the cultivation of strong self-discipline about ethical behaviour, including conforming to strict sexual norms [27]. The implementation of the one-child policy leads to a particularly high level of supervision and monitoring among Chinese children [28]. Compared with their peers in other countries, Chinese children are less likely to experience sexual abuse [28]. However, it must be mentioned that the inconsistency in the data could be partly due to inhibited disclosure stemming from the fear of social stigma and discrimination [29,30]. Traditional Chinese cultural values emphasize that people should abide by social norms and not violate morality, which objectively decreases the prevalence of sexual assault. However, the overvaluing of “chastity” also leads to a reluctance of survivors to talk about their misfortunes and underestimated prevalence. After experiencing sexual harassment, due to sexual shame (reluctance or refusal to talk about one’s sexuality in public) or for reasons of personal future, these survivors choose silence and tolerance [31]. Although we fully informed our respondents that their privacy would be maintained, the impact of this point cannot be ignored. In our study, young male students also had a high prevalence of experiencing sexual assault. There is a common perception that men are rarely sexually assaulted or harassed. [32]. However, recent studies have gradually demonstrated that sexual assault against men is not as uncommon as previously suggested, like dark under the lamp, and has always been ignored by the public [33,34].

Consistent with a large body of findings suggesting that CSA may exert lasting adverse effects on mental health, our study also deduced similar associations and even a dose–response effect [14,35,36]. Different mechanisms may be engaged to interpret the associations between CSA and suicide attempts and mental disorders. Some studies explained the impact of CSA in terms of physiological processes. Blanco indicated that a history of CSA may lead to irregularities in the cortical and subcortical regions of the brain [37]. Guillaume reported that the modulation of hypothalamic–pituitary–adrenal axis (HPA axis) genes altered by child adversity could influence decision making in suicide attempts [38]. Other findings emphasized the social, cultural, and psychological factors below. Sexual abuse might have a dampening impact on the child’s mental health well-being and impair developing capacities for trust and intimacy [39]. The guilt and shame associated with CSA could also become a psychological ticking time bomb, exerting potential and continuous threat to one’s mental and emotional wellbeing [40]. Similarly, social stigma and discrimination could become extrinsic factors that trigger poor psychological conditions, exacerbated by conservative attitudes towards sexuality in Chinese society.

We also noticed that the association between penetrative and contact CSA and suicide attempts was stronger among males than their female counterparts, suggesting that severe CSA might be more detrimental to the mental health of males. These results contradict previous findings that girls were more likely to report a negative reaction to sexual encounters [36,41]. Some studies have pointed out that female trauma survivors are more prone to the use of maladaptive coping mechanisms that often lead to depression, self-harm, and other stress reactions, while men are more likely to remain calm and take criminal action in such incidents [42,43]. However, other studies on populations from Taiwan, mainland China, and Western countries have also supported our conclusions [29,42,44]. First, for a long time, male sexual abuse survivors were ignored because of some stereotypes that men are the dominant perpetrators of sexual assault and could not be sexually abused. It is even widely acknowledged that men revere sexual prowess as a sign of male success and thus regard such unexpected experiences as “getting lucky” and a bragging capital [28]. Indeed, young males also regard aggressive sexual behaviours from females as an unpleasant experience and view it as a form of lack of masculinity and stigma. In addition, under social and cultural perceptions and their own psychological pressure, male survivors will deeply hide this unpleasant experience. Male survivors in China are often questioned as “not man enough” or “too weak” under the definition of masculinity in traditional Chinese culture [42]. Second, society may be more concerned with protecting female members from sexual aggression than males, compounded by the view of females being the weaker sex. Previous studies suggested that approximately 20% of Chinese parents maintained that boys could not possibly be sexually abused [16]. The disclosure of CSA from boys is often doubted and dismissed by their parents [30]. Disappointed and distrustful of their parents or trusted people, the boys abandon all hope of seeking help and psychological support from others. To further aggravate the situation, elementary and secondary education often focus on imparting self-protection knowledge about CSA to girls but overlook sexuality education for boys [45]. When boys experience sexual abuse and assault, they feel overwhelmed and nonplussed. Due to a lack of knowledge of self-protection and limited counselling options, they are more inclined to repress their experience and give up on seeking support and treatment. Given that CSA has more damaging effects on boys than it does on girls, more supporting service and family attention should be placed on male sexual survivors to ensure that their mental well-being is not neglected.

We also found an association between severe CSA and adverse psychological outcomes among college students from rural and suburban areas. To our knowledge, there are only a few studies that have examined on the urban–rural discrepancy between CSA and mental health. The majority of studies have focused on rural-to-urban migrants or rural communities and highlighted the need for child protection in rural China [12,46]. On the one hand, in China, in rural and certain suburban areas, the phenomenon of left-behind children is prevalent, and left-behind children can be affiliated with the absence of parental support because their parents work and live in urban areas, while they remain at home [47]. Previous studies have verified that family support can significantly decrease the likelihood of CSA and alleviate the harmful consequences of CSA among children [48,49,50]. As a result, students from rural and suburban areas were more seriously affected by CSA because they could not receive timely support from their parents. On the other hand, studies have consistently reported rural–urban inequalities in access to education and health care services due to the household registration *(hukou*) system in China [17,51,52]. The household registration (*hukou*) system classified all residents into rural and urban categories based on lineage and place of birth, excluding rural residents from access to state-allocated welfare [53] and controlling migration by restricting individuals’ entitlement to medical insurance and public schooling. Therefore, students from rural and suburban areas with rural *hukou* generally have less education and fewer economic resources than urban students, resulting in less awareness and curricula regarding the threat of CSA; therefore, students inevitably lack important sexuality education, such as the prevention of CSA, and it is difficult to obtain access to qualified mental services after experiencing CSA [12]. Prevention and intervention efforts, such as promoting the accessibility of comprehensive sexuality education, urgently need to be bolstered to target rural and suburban children and adolescents and to reduce the occurrence and detrimental mental implications of CSA.

## 5. Limitations

Despite the many strengths of this study, particularly with regard to the large sample size, the study has the following limitations. First, the cross-sectional design does not allow the definitive establishment of causal inference and only observed associations instead. However, CSA generally antedated most outcomes, suggesting that CSA was a likely predictor and instigator of mental disorders and suicide attempts. Second, self-report data have many limitations, such as recall impediments and memory bias. In China, traditional beliefs call for the suppression of sexuality and place heavy emphasis on the chastity and virginity of women. The data could therefore be subjected to response bias due to social desirability. Third, since the data were obtained from a self-report survey, we cannot ascertain the veracity of CSA cases and outcomes. Moreover, the prevalence of mental disorders might be underestimated without an accurate clinical diagnosis. Further studies should consider combining clinical examinations with interviews and questionnaires. Fourth, the population was limited to college students, so the results cannot be applicable to youths without a college education. Replication with a broader population would be needed for further conclusions. Finally, the spurious relationship between suicide and adverse mental health effects is a highly debatable topic. When experiencing mental trauma, men may prefer to express it in an externalized way, such as suicide. Thus, the psychological effects of CSA on males and females may require further refined research.

## 6. Conclusions

The present research suggested a high prevalence of CSA among Chinese college students of both sexes. It also emphasized that young adult males can be confronted with severe adverse effects on mental health due to CSA. In addition, the influence of urban-rural differences on the associations between CSA and mental health should not be ignored, as college students growing up in rural and suburban environments are often more vulnerable and susceptible to CSA outcomes. Interventions and educational programs should be implemented to prevent the occurrence of CSA and to alleviate its negative consequences. In the meantime, society as a whole should pay more attention to the adverse effects of CSA on males and populations isolated from urbanized areas. Policy-makers and educators should strive to implement and strengthen sexuality education among these vulnerable groups as well as to raise awareness among the general public of the damaging impact of CSA and to change social misconceptions and prejudices towards male survivors.

## Figures and Tables

**Table 1:** Prevalence of childhood sexual abuse among participants by type (*n* = 49,728).

	No (%)			
Variables	Total (*n* = 49,728)	Male (*n* = 16,999)	Female (*n* = 32,729)	*p*-Value
Noncontact CSA				
Experiencing verbal sexual harassment online	12,283 (26.79)	4040 (25.84)	8243 (27.64)	0.0759
Experiencing verbal sexual harassment in real-life situations	13,679 (30.73)	4865 (31.38)	8814 (30.15)	0.2210
Contact CSA				
Being forced to expose one’s private parts (breasts or genitals)	2042 (5.88)	949 (7.16)	1093 (4.74)	<0.0001
Having one’s private parts (breasts or genitals) touched or fondled involuntarily	4210 (10.10)	996 (6.71)	3214 (13.14)	<0.0001
Penetrative CSA				
Being subject to involuntary oral intercourse	592 (1.52)	235 (1.59)	357 (1.46)	0.5914
Being subject to involuntary vaginal or anal intercourse	553 (1.45)	210 (1.50)	343 (1.41)	0.7514
CSA severity				
Non-CSA	30,713 (58.02)	10,698 (60.10)	20,015 (56.16)	<0.0001
Noncontact CSA	13,654 (28.64)	4630 (28.33)	9024 (28.91)	
Contact CSA	4464 (11.07)	1344 (9.37)	3120 (12.60)	
Penetrative CSA	897 (2.27)	327 (2.20)	570 (2.33)	

**Table 2:** Characteristics of respondents without and with a history of different types of CSA (*n* = 49,728).

Variables	Number (Percent) ^a^				
	Total (*n* = 49,728)	Non-CSA (*n* = 30,713)	Noncontact CSA (*n* = 13,654)	Contact CSA (*n* = 4464)	Penetrative CSA (*n* = 897)
Sex					
Male	16,999 (47.14)	10,698 (48.84)	4630 (46.63)	1344 (39.87)	327 (45.70)
Female	32,729 (52.86)	20,015 (51.16)	9024 (53.37)	3120 (60.13)	570 (54.30)
Age					
16–18	16,716 (18.36)	10,926 (20.55)	4440 (16.97)	1129 (12.07)	221 (10.67)
19–21	30,812 (72.77)	18,473 (71.25)	8661 (74.22)	3053 (75.49)	625 (80.00)
22–24	2200 (8.86)	1314 (8.19)	553 (8.81)	282 (12.44)	51 (9.33)
Residential environment					
Rural	11,144 (21.08)	7515 (23.15)	2652 (18.47)	771 (15.85)	206 (26.86)
Suburban	17,107 (32.88)	10,617 (32.84)	4702 (33.01)	1552 (33.13)	266 (30.94)
Urban	21,477 (46.04)	12,581 (44.02)	6300 (48.52)	2171 (51.02)	425 (42.19)
Family structure ^b^					
Complete family	43,215 (85.87)	27,183 (87.07)	11,640 (85.93)	3699 (81.79)	693 (74.01)
The others	6513 (14.13)	3530 (12.93)	2014 (14.07)	765 (18.21)	204 (25.99)
Monthly Expenditure (CNY)					
<1500	25,168 (46.97)	16,555 (50.42)	6410 (44.45)	1821 (37.09)	382 (38.97)
≥1500	24,560 (53.03)	14,158 (49.58)	7244 (55.55)	2643 (62.91)	515 (61.03)
Parental education level ^c^					
Primary school and below	6313 (12.93)	4093 (13.77)	1584 (11.86)	511 (10.82)	125 (15.35)
Middle school/high school	31,828 (63.59)	20,034 (63.99)	8563 (63.61)	2670 (62.11)	561 (60.38)
Vocational school and above	11,003 (22.44)	6117 (20.85)	3422 (23.98)	1262 (26.74)	202 (22.74)
Unknown	584 (1.03)	469 (1.39)	85 (0.55)	21 (0.32)	9 (1.53)
Suicide attempt					
Yes	1779 (3.84)	656 (2.37)	657 (4.47)	365 (7.98)	101 (15.02)
No	47,949 (96.16)	30,057 (97.63)	12,997 (95.53)	4099 (92.02)	796 (84.98)
Mental disorders ^d^					
Yes	3281 (7.74)	1340 (5.24)	1220 (9.78)	578 (13.48)	143 (18.02)
No	46,447 (92.26)	29,373 (94.76)	12,434 (90.22)	3886 (86.52)	754 (81.98)

^a^ Percentages are based on weighted numbers. ^b^ Complete family: no parental divorce, bereavement, or separation during childhood and adolescence. ^c^ The higher education level of parents; the parental education of 574 participants was unknown. ^d^ Mental disorders included depression (5.8%), anxiety (3.3%), bipolar disorder (0.94%), obsessive-compulsive disorder (1.3%), anorexia or bulimia (0.52%), attention deficit hyperactivity disorder (0.18%), phobia (0.58%), posttraumatic stress disorder (0.22%), personality disorders (0.36%), schizophrenia (0.28%), and substance abuse (0.05%).

**Table 3:** Association between mental health and different types of CSA among respondents, stratified by sex (*n* = 49,728).

	Suicide Attempt					Mental Disorders		
	Male		Female		Male		Female	
CSA Severity	Percent (%)	Adjusted OR (95% CI)	Percent (%)	Adjusted OR (95% CI)	Percent (%)	Adjusted OR (95% CI)	Percent (%)	Adjusted OR (95% CI)
Non-CSA (reference group)	1.84	-	2.86	-	5.3	-	5.18	-
Noncontact CSA	1.97	1.06 (0.76–1.50)	6.66	2.08 (1.61–2.69) ***	7.57	1.44 (0.93–2.21)	11.71	2.01 (1.68–2.41) ***
Contact CSA	6.64	3.32 (1.71–6.44) ***	8.87	2.60 (1.90–3.56) ***	11.56	2.04 (1.26–3.31) **	14.74	2.32 (1.77–3.02) ***
Penetrative CSA	13.09	8.15 (3.36–19.74) ***	13.33	3.62 (2.32–5.66) ***	15.99	3.19 (1.53–6.63) **	19.73	3.23 (2.18–4.79) ***

Adjusted for age, hometown, monthly expenditures, family structure, and parents’ education level. Reference group: no CSA. All logistic regression results were weighted. ** *p* < 0.01, *** *p* < 0.001.

**Table 4:** Association between mental health and different types of CSA among respondents, stratified by the residential environment (*n* = 49,728).

	Suicide Attempt						Mental Disorders					
	Rural		Suburban		Urban		Rural		Suburban		Urban	
CSA Severity	Percent (%)	Adjusted OR (95% CI)	Percent (%)	Adjusted OR (95% CI)	Percent (%)	Adjusted OR (95% CI)	Percent (%)	Adjusted OR (95% CI)	Percent (%)	Adjusted OR (95% CI)	Percent (%)	Adjusted OR (95% CI)
Non-CSA (reference group)	1.84	-	1.89	-	2.99	-	4.05	-	4.55	-	6.38	-
Noncontact CSA	2.42	1.30 (0.78–2.15)	3.85	1.97 (1.38–2.81) ***	5.68	1.87 (1.39–2.51) **	4.40	1.02 (0.65–1.60)	6.59	1.40 (1.00–1.95)	13.99	2.22 (1.67–2.95) ***
Contact CSA	5.51	2.96 (1.58–5.54) ***	8.73	3.90 (2.34–6.53) ***	8.26	2.74 (1.95–3.85) ***	8.05	1.77 (0.93–3.35)	13.54	2.58 (1.61–4.14) ***	15.12	2.34 (1.75–3.13) ***
Penetrative CSA	13.73	7.62 (3.18–18.25) ***	18.35	10.01 (3.98–25.22) ***	9.14	2.82 (1.78–4.45) ***	14.80	3.39 (1.41–8.15) **	20.98	5.10 (2.51–10.38) ***	15.70	2.30 (1.54–3.43) ***

Adjusted for age, sex, monthly expenditures, family structure, parent’s education level. Reference group: no CSA. All logistic regression results were weighted. ** *p* < 0.01, *** *p* < 0.001.

## Data Availability

The study data are available upon request from the authors.

## Supplementary Materials

The following supporting information can be downloaded at: https://www.mdpi.com/article/10.3390/ijerph19159225/s1, File S1: Methods.
