# Supplementary material for: Sex and Urban–Rural Differences in the Relationship between Childhood Sexual Abuse and Mental Health among Chinese College Students
Source: Int J Environ Res Public Health. 2022 Jul 28;19(15):9225. doi: 10.3390/ijerph19159225 (PMC9368484; doi:10.3390/ijerph19159225)
Supplement: Supplementary file 1 [file ijerph-19-09225-s001.zip › ijerph-1805331-supplementary.pdf]

Supplementary material: Methods

Methods

Multi-stage sampling

Using a multi-stage sampling, 241 higher learning institutions were selected after balancing the population density, and different levels of educational institutions in China, according to the List of Institutions of Higher Learning released by China's Ministry of Education in 2018.

In Stage 1, all higher education institutions were classified into three categories (eastern, central, and western regions) based on the administrative divisions provided by the National Bureau of Statistics of China.

In Stage 2, select full-time universities and colleges (excluding adult colleges) from each region. Universities are divided into 4 levels: first-class universities, first-class disciplines universities, ordinary universities, and private universities. Colleges are divided into 3 levels: key colleges, ordinary colleges, and private colleges.

In Stage 3, the probability proportional to size (PPS) method was used to select universities and colleges from each level and a total of 241 higher education institutions were selected.

In the final stage, by using the snowball effect of the social network, the questionnaire was sent out to select undergraduates. All the samples are volunteers recruited in the form of convenience sampling. Participation in the survey is decided by the students themselves and can be withdrawn at any time during the process.

Attention Check Questions

Using Attention Check Questions (ACQs) to screen out inattentive respondents. ACQs were included in different parts of the survey, asked participants “You must pay attention to this study. Please tick ‘Don’t know” or “Make sure to select ‘Don’t know’ as an answer so that we know you are paying attention”.
